# Ferroptosis- and stemness inhibition-mediated therapeutic potency of ferrous oxide nanoparticles-diethyldithiocarbamate using a co-spheroid 3D model of pancreatic cancer

**DOI:** 10.1007/s00535-025-02213-3

**Published:** 2025-01-31

**Authors:** Marwa M. Abu-Serie, Ana K. Gutiérrez-García, Macie Enman, Utpreksha Vaish, Huma Fatima, Vikas Dudeja

**Affiliations:** 1 Medical Biotechnology Department, Genetic Engineering and Biotechnology Research Institute, (GEBRI), City of Scientific Research and Technological Applications (SRTA-City), New Borg El‑Arab City, Alexandria, 21934 Egypt; 2 Division of Surgical Oncology, Department of Surgery, University of Alabama at Birmingham (UAB), Birmingham, Alabama, 35294 USA; 3 Department of Pathology, Division of Anatomic Pathology, University of Alabama at Birmingham (UAB), Birmingham, Alabama, 35249 USA

**Keywords:** Pancreatic ductal adenocarcinoma, FeO nanoparticles-diethyldithiocarbamate, Ferroptosis, Stemness inhibition, Pancreatic cancer stem and stellate cells

## Abstract

**Background:**

Pancreatic ductal adenocarcinoma (PDAC) is an aggressive disease with a high mortality rate and exhibits a limited response to apoptosis-dependent chemotherapeutic drugs (e.g., gemcitabine, Gem). This is mainly attributed to the antioxidant defense system (glutathione and aldehyde dehydrogenase (ALDH) 1A1), which sustains stemness features of cancer stem cells (CSCs) and activated pancreatic stellate cells (PSCs)-generated excess stromal proteins. This dense stroma retards drug delivery.

**Methods:**

This study established co-spheroid model consisting of mouse PDAC cell line (KPC) and PSCs (1:5) to accurately investigate the anti-PDAC activity of nanocomplex of ferrous oxide nanoparticles-diethyldithiocarbamate (FeO NPs-DE), compared to Gem, using in vitro and in vivo 3D models.

**Results:**

In vitro and in vivo co-spheroid models demonstrated higher therapeutic efficacy of FeO NPs-DE than Gem. FeO NPs-DE induced selective accumulation of iron-dependent ferroptosis (non-apoptosis)-generated a lethal lipid peroxidation that was potentiated by DE-mediated glutathione and ALDH1A1 suppression. This led to collapse of stemness, as evidenced by down-regulating CSC genes and p-AKT protein expression. Subsequently, gene and/or protein levels of PSC activators (transforming growth factor (TGF)-β, plasminogen activator inhibitor-1, ZEB1, and phosphorylated extracellular signal-regulated kinase) and stromal proteins (collagen 1A2, smooth muscle actin, fibronectin, and matrix metalloproteinase-9) were suppressed. Moreover, DE of nanocomplex enhanced caspase 3-dependent apoptosis with diminishing the main oncogene, BCL-2.

**Conclusions:**

FeO NPs-DE had a stronger eradicating effect than Gem on primary and metastatic peritoneal PDAC tumors. This nanocomplex-mediated ferroptosis and stemness inhibition provides an effective therapeutic approach for PDAC.

**Supplementary Information:**

The online version contains supplementary material available at 10.1007/s00535-025-02213-3.

## Introduction

Currently, pancreatic ductal adenocarcinoma (PDAC) is the fourth most common cause of cancer-related deaths [1] and is expected to be the second leading cause of cancer-related mortality by 2025. The average survival time is approximately six months, with an overall survival rate of 4%. Although surgery is the only effective treatment, 80% of patients are ineligible for surgical resection due to poor diagnosis and aggressive progression [1]. Beyond these factors, PDAC is also resistant to radiotherapy and chemotherapy (gemcitabine), which is mediated by the tumor stemness characteristics triggered by cancer stem cells (CSCs; tumor initiator cells) and activated pancreatic stellate cells (PSCs). PSCs are the major cellular components of the stroma that represent 50–80% of the tumor volume [2, 3]. PSCs are activated upon interaction with cancer cells via autocrine and paracrine signaling, including transforming growth factor (TGF)-β/phosphatidylinositol 3-kinase (PI3K)/AKT, extracellular signal-regulated kinases (ERK), KRAS (main oncogene in PDAC)/plasminogen activator inhibitor (PAI)1, and ZEB1 [4–8]. These activated cells overexpress matrix metalloproteinase (MMP)9 and secrete excessive amounts of extracellular matrix proteins, including smooth muscle actin (SMA), collagen type 1 (COL)1A2, collagen type III, and fibronectin (FN), generating a dense fibrotic stroma [2, 4, 8]. The desmoplastic stroma acts as an obstruction for successful drug delivery and generates hypoxia and hypovascular conditions, conferring chemoresistance and stemness to PDAC [2, 6].

Accordingly, the three-dimensional (3D) spheroid model of a PDAC cell line and PSCs co-culture most closely resembles clinical tumors, making it ideal for the discovery of new effective anti-PDAC drugs, compared to 2D and mono-spheroid cultures [9]. CSC markers (CD24, CD44, and CD133), PSC activation markers (TGF-β, SMA, COL1A2, FN), and common stemness markers (OCT-4, NANOG, aldehyde dehydrogenase (ALDH)1A1, and ATP-binding cassette (ABC)G2 drug transporter) are expressed more in the co-spheroid model than in the mono-spheroid model [1, 9–11]. Herein, the co-spheroid mouse model was established by co-culturing, at a certain ratio, two main PDAC cellular components (cancer cells “KPC” and PSCs) of stemness and drug resistance, to correctly investigate the anti-PDAC efficacy of diethyldithiocarbamate (DE) and its nanoformulations.

Diethyldithiocarbamate is the active metabolite of an FDA-approved anti-alcoholism drug (disulfiram) and irreversibly inhibits ALDH1A1. The latter is a key enzyme for maintaining stemness by protecting CSCs from oxidative stress, inhibiting apoptosis, and enhancing drug resistance [12, 13]. DE was nanoformulated to improve bioavailability, reduce toxicity, and enhance selective accumulation in tumor tissues. In several studies, nanoformulations of DE with copper oxides and ferrous oxide nanoparticles (Cu_4_O_3_ NPs, Cu_2_O NPs, and FeO NPs), exploiting their high metal chelating activity, exhibited potent anticancer efficacy compared to DE, with enhanced non-apoptotic pathways [14–20]. These non-apoptotic signaling pathways are triggered by accumulated copper-stimulated cuproptosis and accumulated iron-stimulated ferroptosis, causing mitochondrial dysfunction and unregulated lipid peroxidation-dependent macromolecule damage, respectively [21, 22]. In addition to DE-mediated apoptosis, cuproptosis and ferroptosis have been proposed as promising targets for eliminating stemness and preventing drug resistance by collapsing the regulated cellular redox status [14, 15, 18–20]. Previous studies demonstrated that DE-Cu(I) exhibited anti-PDAC activity in cell lines and xenograft animal models, and DE-Cu(II)-loaded hyaluronic acid-decorated liposomes reduced the sphere formation potential of patient pancreatic CSCs [23, 24].

In this study, in vitro anti-PDAC activity (cytotoxicity, anti-migration, and anti-invasive effects) of nanocomplexes of DE was investigated using 2D, 3D mono-spheroid, and the established 3D co-spheroid models and compared with gemcitabine (Gem). The most effective nanocomplex (FeO NPs-DE) had been investigated for its ferroptotic effect-mediated co-spheroid growth inhibition. The efficacy of FeO NPs-DE in reducing the count and area of the treated co-spheroids, as well as its suppressive impact on gene expression of CSC and PSC key markers and ALDH1A1 activity were evaluated. Moreover, in vivo anti-PDAC activity of the most active nanocomplex (FeO NPs-DE) was investigated using an orthotopic 3D co-spheroid model. This was evaluated by comparing tumor size (primary and metastatic peritoneal), histological and immunohistochemical analyses, quantification of stemness and activated PSC markers, determination of activities of caspase 3 and ALDH1A1, and assessment of cellular redox parameters and iron cellular uptake (ferroptosis markers).

## Materials and methods

### Materials

Cell culture reagents and phosphate buffer saline (PBS) were purchased from GIBCO (Grand Island, NY, USA). Sigma-Aldrich (St. Louis, MO, USA) supplied collagenase P (Cat#11,249,002,001), MTT, dimethyl sulfoxide (DMSO), protease, copper chloride, ferric nitrate, copper nitrate, vitamin C, NaOH, primers, thiobarbituric acid, and malondialdehyde tetrabutylammonium salt. Additionally, it provided ferrostatin-1, N-Benzyloxycarbonyl-Val-Ala-Asp(O-Me) fluoromethyl ketone (Z-VAD-FMK), ALDH activity colorimetric assay kit (Cat#MAK082), all-trans-retinal (Cat#R2500), glutathione, and Ellman’s reagent (5,5’-dithio-bis-2(nitro benzoic acid). DE (Cat#20,624-25-3) was obtained from Acros Organics (Morris Plains, NJ, USA). Thermo Fisher Scientific (Waltham, Waltham, MA, USA) supplied chitosan, 4',6-diamidino-2-phenylindole (DAPI), RNeasy MinElute Cleanup kit (Cat#NC9088999), cDNA Reverse Transcription kit (Cat#4,368,814), MagNA Lyser Green Beads (Cat# 50-720-3100), RNeasy Micro Kit (Cat# NC9048854), RIPA lysis buffer (Cat#89,900), and Thermo Scientific™ Pierce ECL Western Blotting Substrate (Cat#34,577). Protein assay dye reagent concentrate (Cat#5,000,002) and 4–20% Mini-PROTEAN TGX™ gel (Cat# 4,561,093) were obtained from Bio-Rad (Hercules, CA, USA). The Light Cycler 480 SYBR Green Kit, gemcitabine hydrochloride (Gem, Cat#HY-B0003), and iron colorimetric assay kit (E-BC-K139-M) were acquired from Roche Diagnostics (Mannheim, Germany), MedChemExpress (Monmouth Junction, NJ, USA), and ElabScience (Houston, TX, US), respectively. Cell Signaling Technology (Danvers, MA, USA) provided primary antibodies, including p-AKT (Thr308, rabbit monoclonal antibody #13,038), AKT (rabbit mAb #4370), ERK1/2 (L34F12 mouse mAb #4696), phospho-p44/42 MAPK (ERK1) (Tyr202)/(ERK2) (Tyr204) (D1H6G) (p-ERK1/2, rabbit mAb #4370), and BCL-2 (50E3, rabbit mAb #2870). SMA primary antibody (ab5694), anti-IgG horseradish peroxidase (HRP)-β-actin (ab49900 [AC-15]), caspase-3 assay (colorimetric) kit (ab39401), and dichlorodihydrofluorescein diacetate (DCF-DA) were acquired from Abcam (Cambridge, MA, USA).

## Methods

### Cancer cell lines and PSCs

Human PDAC cell lines, including PANC-1 (CRL-1469) and MIA PaCa-2 (CRL-1420) were obtained from the American Type Culture Collection (ATCC). Mouse PDAC cell line (KPC) was established by harvesting tumors from adult C57/BL6 female mice with LSL-Kras^G12D/+^; LSL-Trp53^R172H/+^; Pdx-1-Cre tumors. These tumor tissues were cut and enzymatically digested using collagenase IV and DNase I. Following culturing and trypsinization, KPC cells were isolated from other cultured cells using EPCAM, as previously described [25]. PANC-1 “passage no. 25” and KPC “passage no. 12” were cultured in DMEM and DMEM/F12, respectively, containing 10% fetal bovine serum (FBS).

Stellate cells were enzymatically isolated from pancreas tissues of C57/BL6 female mice (6 weeks old) using collagenase P, protease, and DNase1 according to the method of Apte et al. (1998), and the isolated PSCs were cultured in IMEM-containing 10% FBS [26].

### Preparation of DE nanoformulation using metal oxide NPs

**Fig. 1:**
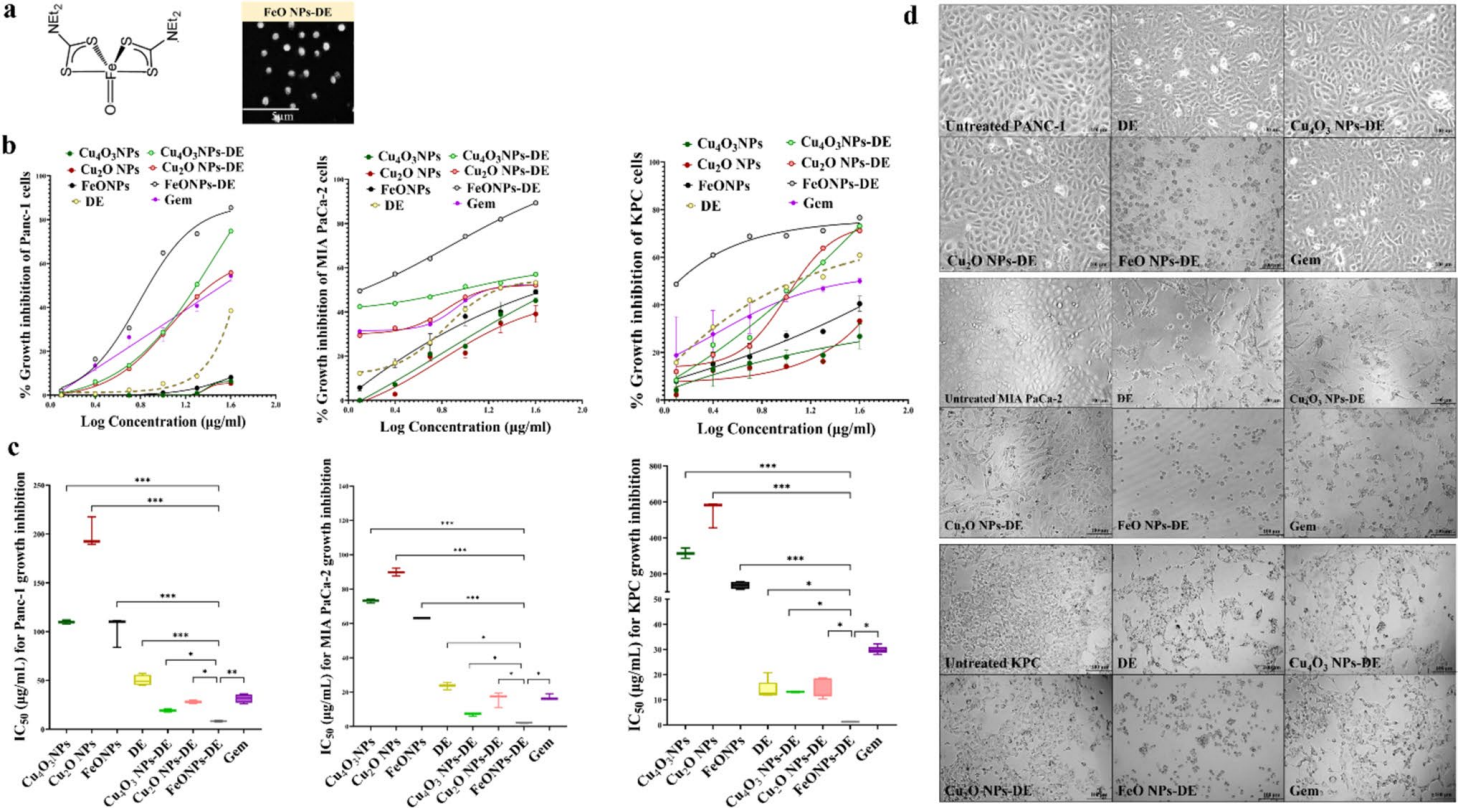
Structure and morphology of FeO nanoparticles (NPs)-diethyldithiocarbamate (DE) as well as MTT results for in vitro cytotoxicity against pancreatic cancer cell lines (2D model). **a** 2D structure and scanning electron microscopy images of FeO NPs-DE (5000 × magnification). Growth inhibition of human Panc-1, human MIA PaCa-2, and mouse KPC cells after 24 h treatment with metal oxide nanoparticles (Cu_4_O_3_ NPs, Cu_2_O NPs, and FeO NPs), DE, nanocomplexes (Cu_4_O_3_ NPs-DE, Cu_2_O NPs-DE, and FeO NPs-DE), and standard chemotherapy (gemcitabine, Gem), as illustrated by **b** dose–response curves and **c** half-maximal inhibitory concentration (IC_50_) values, as well as **d** morphological variations of the treated Panc-1, MIA PaCa-2, and KPC cell lines (100 × magnification). Data presented as mean ± SEM. The nanocomplex of FeO NPs-DE was compared to other compounds and values are considered statistically significant at *p* < 0.05*, < 0.001**, and < 0.0001***

As recently described, Cu_4_O_3_ NPs or Cu_2_O NPs were obtained by mixing 16 mM copper chloride or copper nitrate, respectively, with 2% chitosan and 20% vitamin C and centrifuging the mixture [14, 17]. For the preparation of FeO NPs, 160 mM Fe (NO_3_)_3_^.^9H_2_O and vitamin C (20%) were added to 1 N NaOH and then a black pellet was harvested after centrifugation [16]. As demonstrated in previous studies, these metal oxide NPs with sizes of 112.5, 111.6, and 38.8 nm, respectively, were characterized by energy-dispersive X-ray analysis, X-ray diffractometry, and electron microscopy. DE was nanoformulated by mixing DE separately with the metal oxide NPs at a ratio of 1:0.1 [14, 16, 17]. The 2D structure of the most active nanocomplex (FeO NPs-DE) was drawn using ChemDraw v.16.0 (Fig. 1a) as well as its morphology was investigated using scanning electron microscopy (SEM).

### Investigation of anti-pancreatic cancer activity using the 2D model

#### Cytotoxicity testing in human and mouse pancreatic cancer cell lines

In 96-well cell culture plates, 5 × 10^3^ PDAC cells (PANC-1, MIA PaCa-2, or KPC) were seeded per well and incubated with serial concentrations of metal oxide NPs, DE, nanocomplexes (Cu_4_O_3_ NPs-DE, Cu_2_O NPs-DE, or FeO NPs-DE), or Gem after cell attachment. Following 24 h, 20 µL MTT (5 mg/mL) was incubated for 4 h with the untreated and treated cells, and then DMSO was added and absorbances were measured at 590 nm [27]. The growth inhibition percentages in both treated cell lines were estimated to calculate growth inhibitory concentration at 50% (IC_50_) using GraphPad Prism 9. Morphological alterations were recorded using a phase contrast microscope (Fisher Scientific, Waltham, MA, USA).

#### Determination of anti-migration activity

Before assessing the anti-migration potential, the maximum safe doses of the most active compounds (DE and its nanocomplexes) and standard chemotherapy for the viability of KPC cells were determined. Then, 100 µL of 0.2 × 10^6^ KPC cells in serum-free DMEM/F12 medium containing the safe dose (0.2 µg/mL) of DE, nanocomplexes, or Gem was added to the insert well of a 24-transwell plate with a polyethylene therephthalate membrane (8 µm pore size). The untreated and treated inserts were then transferred to wells containing 750 µL of culture medium. After 12 h of incubation, the insert wells were washed twice with PBS, fixed with 4% paraformaldehyde, washed twice, incubated with 0.1% triton, washed twice, and stained with DAPI. After scraping the unmigrated cells and washing, the migrated cells were imaged using a fluorescence microscope (Life Technologies, Carlsbad, CA, USA) and counted using Image J software.

### Investigation of anti-pancreatic cancer activity using mono-spheroid 3D model

#### Cytotoxicity testing in human and mouse pancreatic cancer spheroids

Pancreatic cancer cell lines (PANC-1, MIA PaCa-2, or KPC) and PSCs were suspended in a culture medium containing a 0.25% methylcellulose solution that was prepared as previously described. Briefly, 6 g of autoclavable methylcellulose was dissolved in 250 mL preheated basal medium, mixed overnight with 250 mL culture medium with 20% FBS, and centrifuged to collect the supernatant [28, 29]. This hypoxic medium enhanced spheroid formation. These PDAC cells (1 × 10^4^ /well) were seeded in ultra-low attachment 96-well plates. After 72 h, the generated spheroids were incubated with serial dilutions of DE, nanocomplexes (Cu_4_O_3_-DE NPs, Cu_2_O NPs-DE, or FeO NPs-DE), and Gem for 72 h. Then, 20 µL of MTT (5 mg/mL) was incubated for 4 h with the untreated and treated co-spheroids, and then DMSO was added and the absorbances were measured at 590 nm after overnight incubation [27]. IC_50_ values were estimated by GraphPad Prism 9 and morphological variations were recorded utilizing a phase contrast microscope (Fisher Scientific, Waltham, MA, USA).

#### Determination of the impact on stemness gene expression, ALDH1A1 activity, and lipid peroxidation content in the treated human and mouse PDAC mono-spheroids

After 72 h treatment of Panc-1 spheroids and KPC spheroids with the most active nanocomplex (FeO NPs-DE) or Gem, spheroids were harvested, trypsinized, and lysed for RNA extraction and cDNA synthesis using RNeasy MinElute Cleanup kit and the cDNA Reverse Transcription kit. The Light Cycler 480 SYBR Green kit and specific primers of stemness genes (Supplementary Table 1 and supplementary Table 2) were then used for the qPCR assay.

For determination of the inhibition in ALDH1A1 activity, the untreated and treated mono-spheroids were lysed with the kit’s lysis buffer, centrifuged, and the obtained supernatants were added to the substrate mix reagent and all-*trans*-retinal (substrate). The change in absorbance (0 and 15 min) was measured at 405 nm to estimate ALDH1A1 activity using the NADH standard curve. The percentage of ALDH1A1 inhibition was detected in comparison to the untreated spheroids.

Cellular lipid peroxidation level (hall-marker of ferroptosis) was determined by incubating the supernatants of the untreated and treated mono-spheroids with 0.67% thiobarbituric acid at 95°C for 60 min [30]. After centrifugation, the supernatants were harvested and measured at 532 nm. The lipid peroxidation product concentration was estimated using a standard curve of malondialdehyde, normalized to the cellular protein content detected by the protein assay dye reagent concentrate, and calculated relative to the untreated wells.

### In vitro investigation of anticancer activity using the KPC:PSC co-spheroid 3D model

#### Establishment of the KPC:PSC co-spheroid model

To optimize the KPC:PSC ratio, the most aggressive feature (invasion) was assessed for various ratios (1:1, 1:3, 1:5, 1:7, and 1:9) in comparison with the individual KPC and PSC mono-spheroids. Both cells alone or in co-culture were suspended in a culture medium containing the forementioned 0.25% methylcellulose solution. For single spheroid generation, the hanging drop method was used by pipetting 25 µL of 1000 cells of each individual type at different ratios of co-culture on the inner sides of the Petri dish lids, and the Petri dish plates were filled with 13 mL of PBS. After 72 h, each spheroid was harvested, resuspended in pre-chilled Matrigel (diluted 30 times in basal medium), and seeded into a 12-well cell culture plate. After incubation for 45 min in a CO_2_ incubator for solidification, 2 mL of culture medium was added. After 72 h, the invasion area of each mono-spheroid or co-spheroid was estimated using ImageJ software.

The co-spheroid (1:5), which had the highest invasion area, was prepared in a 75 cm^2^ ultra-low-attachment T-flask. Furthermore, KPC, PSC, and KPC:PSC (1:1) spheroids were prepared for comparison with KPC:PSC (1:5) spheroids in term of gene expression of stemness and activated PSCs, including CD24, CD44, CD133, ATP-binding cassette (ABC)G2, ALDH1A1, OCT-4, TGF-β, COL1, SMA, FN, hypoxia-inducible factor (HIF)-1α, and MMP9. The harvested spheroids and co-spheroids were trypsinized and the RNA was extracted for cDNA synthesis using the above-mentioned kits. The qPCR assay was then performed using the Light Cycler 480 SYBR Green kit and specific primers (Supplementary Table 2).

#### Investigation of cytotoxicity and ferroptosis-mediated growth inhibition in the treated KPC:PSC co-spheroids

In ultra-low-attachment 96-well plates, 50 µL of KPC (2000 cells) and 50 µL of PSC (1 × 10^4^ cells) in 0.25% methylcellulose-containing culture medium were seeded per well. After 72 h of co-spheroid formation, serial concentrations of metal oxide NPs, DE, nanocomplexes (Cu_4_O_3_-DE NPs, Cu_2_O NPs-DE, or FeO NPs-DE), and Gem were added. The spheroid viability was assessed using the MTT assay [27], as described above. IC_50_ values were calculated using GraphPad Prism 9 and morphological changes were documented using a phase contrast microscope (Fisher Scientific, Waltham, MA, USA). Additionally, the co-spheroid number and area were estimated in Gem-treated wells and the most active nanocomplex (FeO NPs-DE)-treated wells relative to untreated wells.

To examine ferroptosis as the primary cause of spheroid death, co-spheroids were pre-treated with 1 µM ferrostatin-1 (ferroptosis inhibitor) or 10 µM Z-VAD-FMK (apoptosis inhibitor) for 24 h [31] before incubating with FeO NPs-DE at various concentrations for 72 h. The percentages of spheroid growth inhibition were determined by applying the above-mentioned MTT protocol as well as the morphological variations in the treated co-spheroids were observed utilizing a phase contrast microscope (Fisher Scientific, Waltham, MA, USA).

#### Determination of the impact on co-spheroid gene expression, invasion and lipid peroxidation

In ultra-low-attachment T75 flasks, FeO NPs-DE or Gem (74 µg/mL) was incubated with the co-spheroids for 72 h, and then the untreated and treated co-spheroids were collected for RNA extraction, cDNA synthesis, and qPCR assays to determine the relative changes in the expression of the aforementioned genes.

To evaluate the anti-invasion activity, as already described, the hanging drop protocol was used to prepare individual co-spheroids that were resuspended in diluted Matrigel, seeded, and cultured in a medium containing the maximum safe dose (3 µg/mL) of FeO NPs-DE or Gem. After 72 h, the invasion area in the treated wells was calculated relative to that in the untreated wells.

Briefly, the lipid peroxidation level was quantified using the aforementioned thiobarbituric acid method [30]. The fold increase in the lipid peroxidation level of the treated co-spheroids was estimated in comparison with the untreated co-spheroids.

### In vivo investigation of anti-PDAC activity using the established spheroid model (KPC:PSC co-spheroids)

#### Experimental design

This is the first study utilizing KPC:PSC (1:5 ratio) co-spheroids for in vivo induction of PDAC, so the co-spheroid number requires standardization. The co-spheroid number was adapted from preliminary trials injecting 10, 50, or 100 spheroids/mouse, and the tumors formed in each group (5 mice/group) were harvested, weighed, and investigated histologically.

Female C57/BL6 mice (7 weeks old, weight 20–25 g) were anesthetized by intraperitoneal injection of a mixture of 10% ketamine (100 mg/kg) and 1% xylazine (10 mg/kg). Subsequently, 500 µL of saline and 20 µL of buprenophine (1 mg/kg) were administered subcutaneously for hydration and pain control, respectively. The pancreatic tumor was orthotopically induced by injecting KPC:PSC co-spheroids (100 spheroids/10 µL cold Matrigel/mouse) into the pancreas, using a Hamilton syringe with a 22-gauge needle. After one week, 21 mice were randomly divided into three groups (PBS-treated, FeO NPs-DE-treated, and Gem-treated), which were injected intraperitoneally with PBS, FeO NPs-DE (50 mg/kg body weight), and Gem (50 mg/kg body weight) [32], respectively, three times weekly. Following treatment for three weeks, the mice were sacrificed and tumor tissues and other tissues were harvested. The primary pancreatic tumor and peritoneal metastasized tumor burdens were weighed. Primary tumor tissues were divided into two parts: a smaller portion was fixed in 10% formalin for histological and immunohistochemical investigations, and the other portion was stored at -80°C for molecular and biochemical analyses. The normal parts of the pancreas, liver, brain, lungs, and kidneys were fixed in 10% formalin for histological analysis.

#### Histological analysis and qPCR detection of CSC and the activated PSC key genes

Fixed paraffin-embedded tumor tissue slides were stained with hematoxylin and eosin (H&E) following a typical procedure.

For the qPCR assay, MagNA Lyser Green beads and a MagNA Lyser instrument (Roche Diagnostics, Indianapolis, IN, USA) were used to homogenize tumor tissues, and the supernatants were collected for RNA extraction using the RNeasy Micro Kit. The cDNA Reverse Transcription kit was used to prepare cDNA. The cDNA samples were then used with the Light Cycler 480 SYBR green kit and specific primers (Supplementary Table 1) to detect relative changes in the gene expression of CD24, CD44, CD133, ABCG2, ALDH1A1, NANOG, NOTCH1, OCT-4, SOX2, TGF-β, PAI, COL1A2, SMA, FN, HIF-1α, and MMP9 using the Eq. 2^−ΔΔCT^.

#### Western blot analysis for p-AKT/AKT, p-ERK/ERK, and SMA

Briefly, tumor tissues were homogenized in protease inhibitor- and phosphatase inhibitor-containing RIPA buffer to extract proteins that were quantified using a protein assay dye reagent concentrate. After protein denaturation, loading on 4–20% Mini-PROTEAN TGX™ gel, and transferring onto nitrocellulose membranes, all blots were blocked with 5% skimmed milk. The blots were washed and incubated overnight with 1:1000 diluted antibody against p-AKT**,** AKT, ERK1/2, p-ERK1/2, and SMA. After washing and incubation with 1:2000 anti-IgG, a substrate solution was added and bands were visualized using a chemiluminescent imaging system (ImageQuant™ LAS 500; GE Healthcare Bio-Sciences, Piscataway, NJ, USA). All blots were washed and incubated with HRP-β-actin, after which the substrate working solution was added and bands were recorded using a chemiluminescent imaging system (ImageQuant™ LAS 500; GE Healthcare Bio-Sciences, Piscataway, NJ, USA). Protein expression levels were normalized to their corresponding β-actin levels using ImageJ software. p-AKT and p-ERK levels were estimated as ratios of total AKT and total ERK levels, respectively. The ratios and SMA values of the treated mouse groups were calculated relative to those of the PBS-treated group.

#### Determination of caspase 3 activity and relative BCL-2 protein level

Briefly, caspase 3 activity was assessed in tumor tissues after homogenization in the lysis buffer and centrifugation. Supernatants were mixed with the reaction buffer and incubated with the substrate for 2 h at 37 °C, as described in the caspase-3 assay (colorimetric) kit. The absorbances of the product (chromophore p-nitroaniline) were then measured at 405 nm using a SpectraMax microplate reader (Marshall Scientific, Hampton, NH, USA).

The standard immunohistochemistry (IHC) protocol was used to detect the protein level of BCL-2 in the fixed paraffin tumor sections of the treated mice, relative to the PBS-treated group, by staining with 1:100 anti-BCL-2.

#### Determination of ferroptosis (accumulative iron level and oxidative stress) markers with assessment of ALDH1A1 inhibition

After homogenizing tissues in PBS, centrifugation, and collection of supernatants, the tumoral uptake of FeO NPs-DE was evaluated by determining the equivalent iron in tumor tissues and comparing it to the normal part of the pancreas and other normal tissues. This procedure was carried out in accordance with the manufacturer’s instructions for the iron colorimetric assay kit.

To detect other parameters, tumor tissues were homogenized in chilled PBS and centrifuged. According to previously described methods [16, 33], the GSH level was quantified by incubation with Ellman’s reagent. GPX4 activity was measured colorimetrically using methods established in previous studies [15, 34]. The ROS content was determined by incubation with DCF-DA. The fluorescence product (DCF) level, equivalent to the ROS content, was assessed at 485 nm (excitation) and 535 nm (emission) using a fluorescence microplate reader (Marshall Scientific, Hampton, NH, USA). The ROS level was calculated using a standard curve of t-butyl hydroperoxide. The lipid peroxidation level was assessed applying thiobarbituric acid reactive substance assay, as described above [30]. 

The inhibition of ALDH1A1 activity was assessed in tumor tissues after homogenization in lysis buffer using the forementioned ALDH activity colorimetric assay kit. In all of the aforementioned assays, values were normalized to protein levels, and data obtained from the treated mouse group were calculated relative to the PBS-treated group.

#### Histological analysis of normal tissues

Following the conventional procedure, fixed paraffin tissue slides of the pancreas (normal part), liver, brain, lung, and kidney were stained with hematoxylin and eosin (H&E) to investigate the toxicity of FeO NPs-DE and Gem on normal tissues.

### Statistical analysis

Data, presented as the mean ± standard error of the mean (SEM), were analyzed by one-way analysis of variance (ANOVA) multiple comparisons with Tukey's post hoc test as well as the t-test using GraphPad Prism 9.3.1. The thresholds for statistical significance were set at *p* ≤ 0.05, ≤ 0.005**, and ≤ 0.001***.

## Results

Diethyldithiocarbamate was nanoformulated by mixing with the characterized metal oxide NPs to form nanocomplexes of Cu_4_O_3_ NPs-DE, Cu_2_O NPs-DE, or FeO NPs-DE (semi-circular shape as demonstrated by SEM (Fig. 1a)), with sizes of 156.5 nm, 148.1 nm, and 157.8 nm, respectively [14, 16, 17].

### Powerful anticancer activity of FeO NPs-DE against pancreatic cancer cell lines (2D model) and mono-spheroids (3D model)

**Fig. 2:**
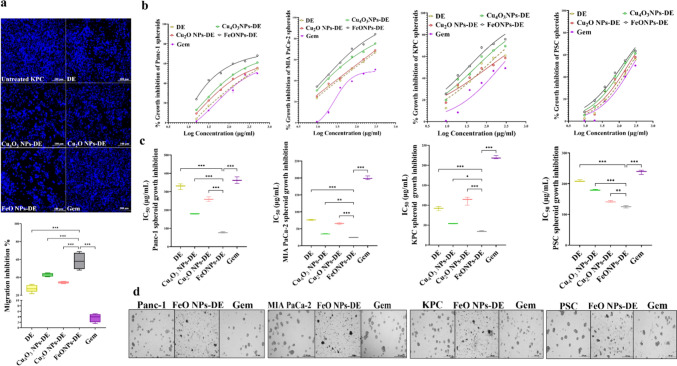
Anti-migration potential of diethyldithiocarbamate (DE) and its nanocomplexes in pancreatic cancer cells (2D model) as well as in vitro cytotoxicity against pancreatic cancer mono-spheroids (3D model). **a** Anti-migration activity in the treated KPC cells as demonstrated by microscopic images of DAPI-stained migratory cells (100 × magnification) and the percentage of migration inhibition. Growth inhibition of human Panc-1, human MIA PaCa-2, and mouse KPC spheroids, as well as pancreatic stellate (PSC) spheroid by metal oxide nanoparticles (Cu_4_O_3_ NPs, Cu_2_O NPs, and FeO NPs), DE, nanocomplexes (Cu_4_O_3_ NPs-DE, Cu_2_O NPs-DE, and FeO NPs-DE), and standard chemotherapy (gemcitabine, Gem), as illustrated by **b** dose–response curves and **c** half-maximal inhibitory concentration (IC_50_) values, as well as **d** morphological variations of the treated Panc-1, MIA PaCa-2, and KPC cell lines (100 × magnification). The nanocomplex of FeO NPs-DE was compared to other compounds and values are considered statistically significant at *p* < 0.05*, < 0.001**, and < 0.0001***

In a dose-dependent manner, these prepared nanocomplexes (IC_50_ ≥ 1.23 µg/mL) exhibited higher cytotoxicity than Gem (IC_50_ ≥ 17 µg/mL) against Panc-1, MIA PaCa-2, and KPC cells, the corresponding metal oxide NPs (IC_50_ ≥ 63 µg/mL), and DE (IC_50_ > 14 µg/mL). It is worth mentioning that FeO NPs-DE had the lowest IC_50_ (8.21, 2.01, and 1.23 µg/mL) compared to other nanocomplexes of Cu_4_O_3_ NPs-DE (19.1, 7.11, and 13.12 µg/mL) and Cu_2_O NPs-DE (27.9, 16.1, and 13.86 µg/mL) against Panc-1, MIA PaCa-2, and KPC cells, respectively (Fig. 1b,c). As illustrated in Fig. 1d, FeO NPs-DE-treated Panc-1, MIA PaCa-2, and KPC cells showed severe collapse in their morphology compared to other treated cells. Moreover, FeO NPs-DE showed the strongest anti-migration activity (58.2 ± 4.6%), evidenced by a decrease in the number of migrated DAPI-labelled cells, compared to the other nanocomplexes, DE, and Gem (Fig. 2a).

Importantly, this active nanocomplex had the strongest inhibitory potency on the growth of pancreatic cancer mono-spheroids (Panc-1, MIA PaCa-2, and KPC) in a dose–response manner (Fig. 2b). This was evidenced by its lowest IC_50_ (77.8, 24.3, and 34.4 µg/mL, respectively) compared to DE and other nanocomplexes (> 77, > 34, and > 53 µg/mL, respectively), as well as extreme collapse in FeO NPs-DE-treated spheroid shapes (Fig. 2c,d). DE, DE-copper oxide nanocomplexes, FeO NPs-DE, and Gem revealed higher IC_50_ (209, > 142, 125, and 238 µg/mL, respectively) for inhibition of PSC spheroid growth than pancreatic cancer spheroids, indicating their low cytotoxicity against PSC spheroids. Based on the IC_50_ value (125 µg/mL) and morphological damage in FeO NPs-DE-treated PSC spheroids, FeO NPs-DE was the strongest inhibitor of PSC spheroid growth (Fig. 2b–d).

**Fig. 3:**
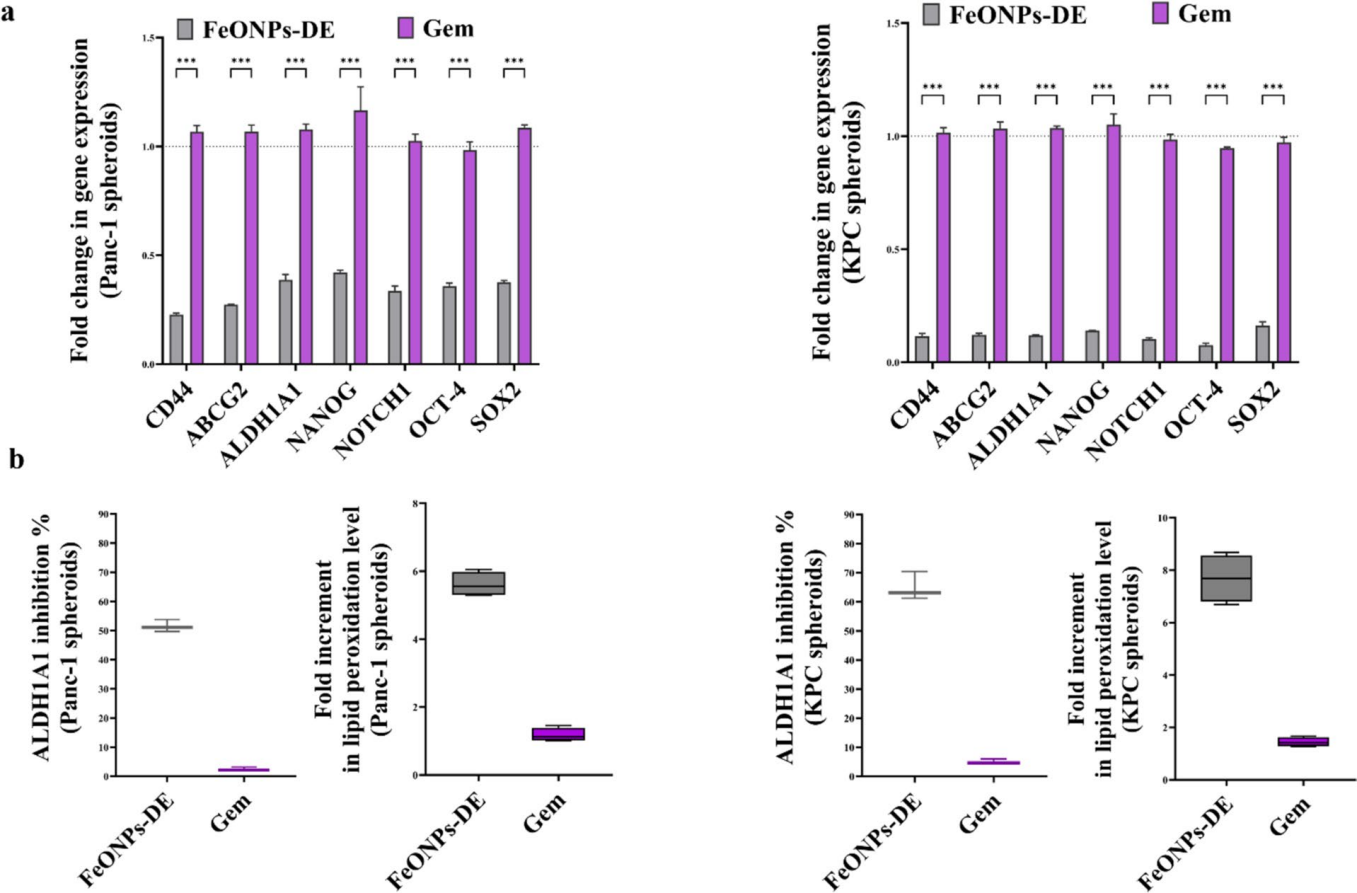
Inhibition of the stemness gene expression and ALDH1A1 activity as well as elevation of lipid peroxidation in the treated human and mouse pancreatic cancer mono-spheroids (3D model). **a** Relative fold change in the expression of the main stemness genes (CD44, ATP-binding cassette drug transporter (ABC)G2, aldehyde dehydrogenase (ALDH)1A1, NANOG, NOTCH1, OCT-4, and SOX2) in the most active nanocomplex (FeO nanoparticles (NPs)-diethyldithiocarbamate (DE))- and standard chemotherapy (gemcitabine, Gem)-treated Panc-1 spheroids and -treated KPC spheroids, relative to the untreated corresponding spheroids. **b** The inhibition percentage of ALDH1A1 activity and relative fold increase in lipid peroxidation level in the treated Panc-1 and KPC spheroids. Data presented as mean ± SEM. The nanocomplex of FeO NPs-DE was compared to Gem and values are considered statistically significant at *p* < 0.05*, < 0.001**, and < 0.0001***

Moreover, the most active nanocomplex (FeO NPs-DE) quelled significantly the key stemness genes (CD44, ABCG2, ALDH1A1, NANOG, NOTCH1, OCT-4, and SOX2) by ≥ 2.3 folds and ≥ 5.5 folds, compared to Gem, in the treated Panc-1 spheroids and KPC spheroids with its corresponding IC_50_, respectively (Fig. 3a). Its stemness suppressive activity was also affirmed by a higher inhibition effect on ALDH1A1 by 51.52% and 64.93% in the treated Panc-1 spheroids and KPC spheroids, respectively, compared to ≤ 6% in Gem-treated human and mouse PDAC mono-spheroids (Fig. 3b). Besides, FeO NPs-DE-treated Panc-1 spheroids and KPC spheroids showed 5.6-fold and 7.7-fold elevations in lipid peroxidation levels, respectively, compared to Gem-treated PDAC mono-spheroids (Fig. 3b).

### Promising influence of FeO NPs-DE on growth, gene expression, and redox status of KPC:PSC spheroids (in vitro 3D model)

**Fig. 4:**
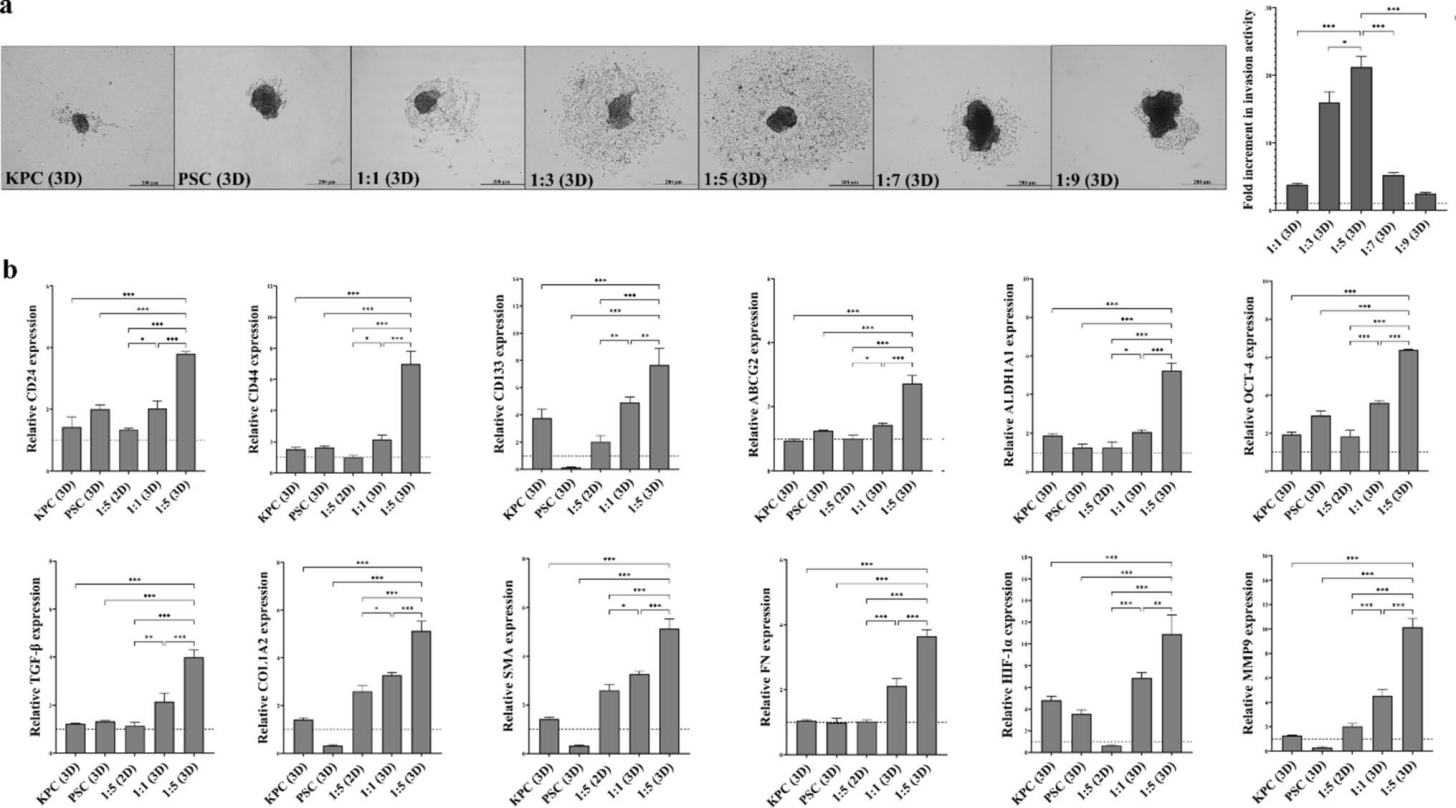
Invasion activities and key gene expression levels in KPC, PSCs, and co-spheroids. **a** Invasion potentials of KPC mono-spheroids, PSC mono-spheroids, and different ratios of KPC:PSC co-spheroids as demonstrated by microscopic images (40 × magnification) and fold increment in invasion activity. **b** Relative fold increment in the expression of CD24, CD44, ATP-binding cassette drug transporter (ABC)G2, aldehyde dehydrogenase (ALDH)1A1, OCT-4, transforming growth factor (TGF)-β, collagen (COL)1A2, smooth muscle actin (SMA), fibronectin (FN), and matrix metalloproteinase (MMP)9 in KPC mono-spheroids, PSC mono-spheroids, 1KPC:5PSC coculture 2D model, 1KPC:1PSC co-spheroids, and 1KPC:5PSC co-spheroids. Data presented as mean ± SEM. 1KPC:5PSC co-spheroids were compared to other cells and spheroids. Values are considered statistically significant at *p* < 0.05*, < 0.001**, and < 0.0001***

As PSC is one of the major components of the stroma, which represents a large volume of pancreatic tumor, a spheroid 3D model was prepared by increasing the proportion of PSC to KPC, generating co-spheroids. Based on the PSC spheroids having approximately five times the area of KPC spheroids, the area of the formed co-spheroids increased as the KPC:PSC ratio increased; therefore, the 1KPC:9PSC co-spheroid had the largest area (Supplementary Fig. 1a). To select the co-spheroid with the most aggressive tumorigenic features, the invasion area of each co-spheroid was compared with that of the KPC spheroid. Figure 4a shows that 1KPC:5PSC co-spheroid had the highest invasion activity, which was 21-fold and ≥ twofold relative to the KPC mono-spheroid and other co-spheroids, respectively. The morphology of the 2D and 3D models of KPC, PSC, and 1KPC:5PSC was recorded using a phase-contrast microscope (Supplementary Fig. 1b). Compared with KPC spheroids, PSC spheroids, 1KPC:1PSC spheroids, and the 1KPC:5PSC co-culture 2D model, the 1KPC:5PSC spheroids exhibited significantly the highest expression of stemness genes (ALDH1A1, CD24, CD44, CD133, OCT-4, and ABCG2), PSC genes (TGF-β, COL1, SMA, and FN), HIF-1α, and MMP9 (Fig. 4b). Accordingly, 1KPC:5PSC was the best representative spheroid 3D model for assessing the anticancer activity of the studied compounds.

**Fig. 5:**
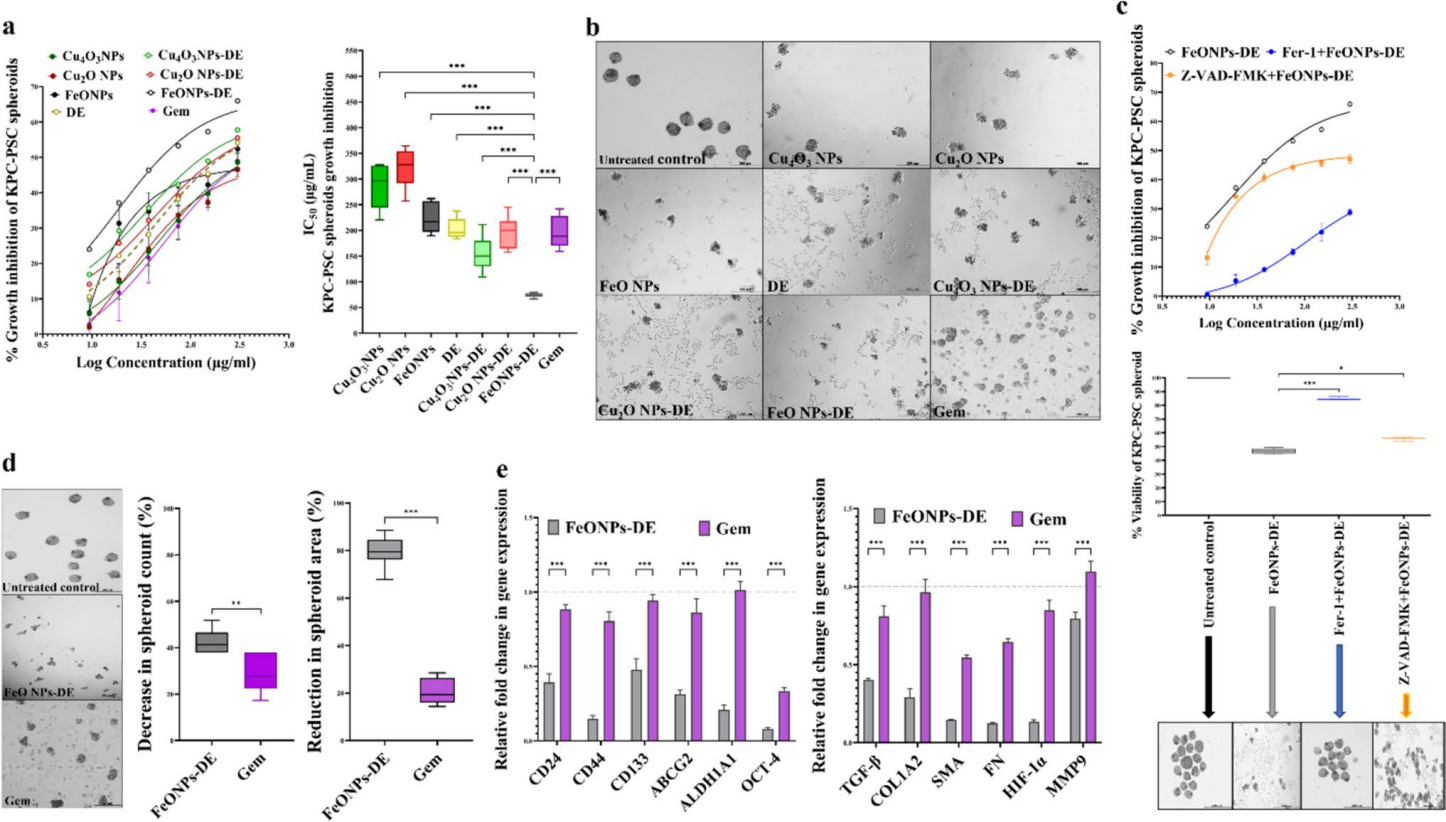
Cytotoxicity, investigation of ferroptosis/apoptosis-mediated growth inhibition, and gene expression changes in the treated 1KPC:5PSC co-spheroids (targeted 3D model of pancreatic cancer). **a** Growth inhibition effect of metal oxide nanoparticles (Cu_4_O_3_ NPs, Cu_2_O NPs, and FeO NPs), diethyldithiocarbamate (DE), nanocomplexes (Cu_4_O_3_ NPs-DE, Cu_2_O NPs-DE, and FeO NPs-DE), and standard chemotherapy (gemcitabine, Gem), as shown by dose–response curve and half-maximal inhibitory concentration (IC_50_) values, as well as **b** morphological changes (40 × magnification). **c** Dose–response curve for the growth inhibition % of co-spheroid after preincubation with 1 µM ferrostatin-1 (Fer-1, ferroptosis inhibitor) or 10 µM Z-VAD-FMK (apoptosis inhibitor) and subsequent treatment with serial concentrations of FeO NPs-DE, as well as percentages of co-spheroid viability and their morphological modifications at 1 µM Fer-1 + 74 µg/mL “IC_50_” FeO NPs-DE and 10 µM Z-VAD-FMK + 74 µg/mL FeO NPs-DE, relative to the untreated co-spheroids and FeO NPs-DE-treated co-spheroids, showing ferroptosis as the major mediator of spheroid death (MTT assay). Data presented as mean ± SEM. The nanocomplex of FeO NPs-DE was compared to other compounds and values are considered statistically significant at *p* < 0.05*, < 0.001**, and < 0.0001***. **d** Inhibitory impact on morphology, count, and area, as well as **e** downregulation of gene expression of CD24, CD44, ATP-binding cassette drug transporter (ABC)G2, aldehyde dehydrogenase (ALDH) 1A1, OCT-4, transforming growth factor (TGF)-β, collagen (COL)1A2, smooth muscle actin (SMA), fibronectin (FN), and matrix metalloproteinase (MMP)9 in the treated co-spheroids. Data presented as mean ± SEM. The nanocomplex of FeO NPs-DE was compared to Gem and values are considered statistically significant at *p* < 0.05*, < 0.001**, and < 0.0001***

According to IC_50_ values, FeO NPs-DE (73.9 µg/mL) exhibited the maximum cytotoxicity on 1KPC:5PSC co-spheroids, compared to metal oxide NPs (≥ 223 µg/mL), DE (204 µg/mL), other nanocomplexes (≥ 154 µg/mL), and Gem (195 µg/mL) in a dose-dependent manner (Fig. 5a). Furthermore, extreme morphological alterations in the FeO NPs-DE-treated co-spheroids confirmed their cytotoxic potency (Fig. 5b). As shown in Fig. 5c, preincubating FeO NPs-DE (18.7–300 µg/mL)-treated co-spheroids with the ferroptosis inhibitor (Fer-1) arrested growth inhibition by > 71%, compared to < 66% in the case of the apoptosis inhibitor (Z-VAD-FMK), declaring that the induced cell death is mostly mediated by ferroptosis.

**Fig. 6:**
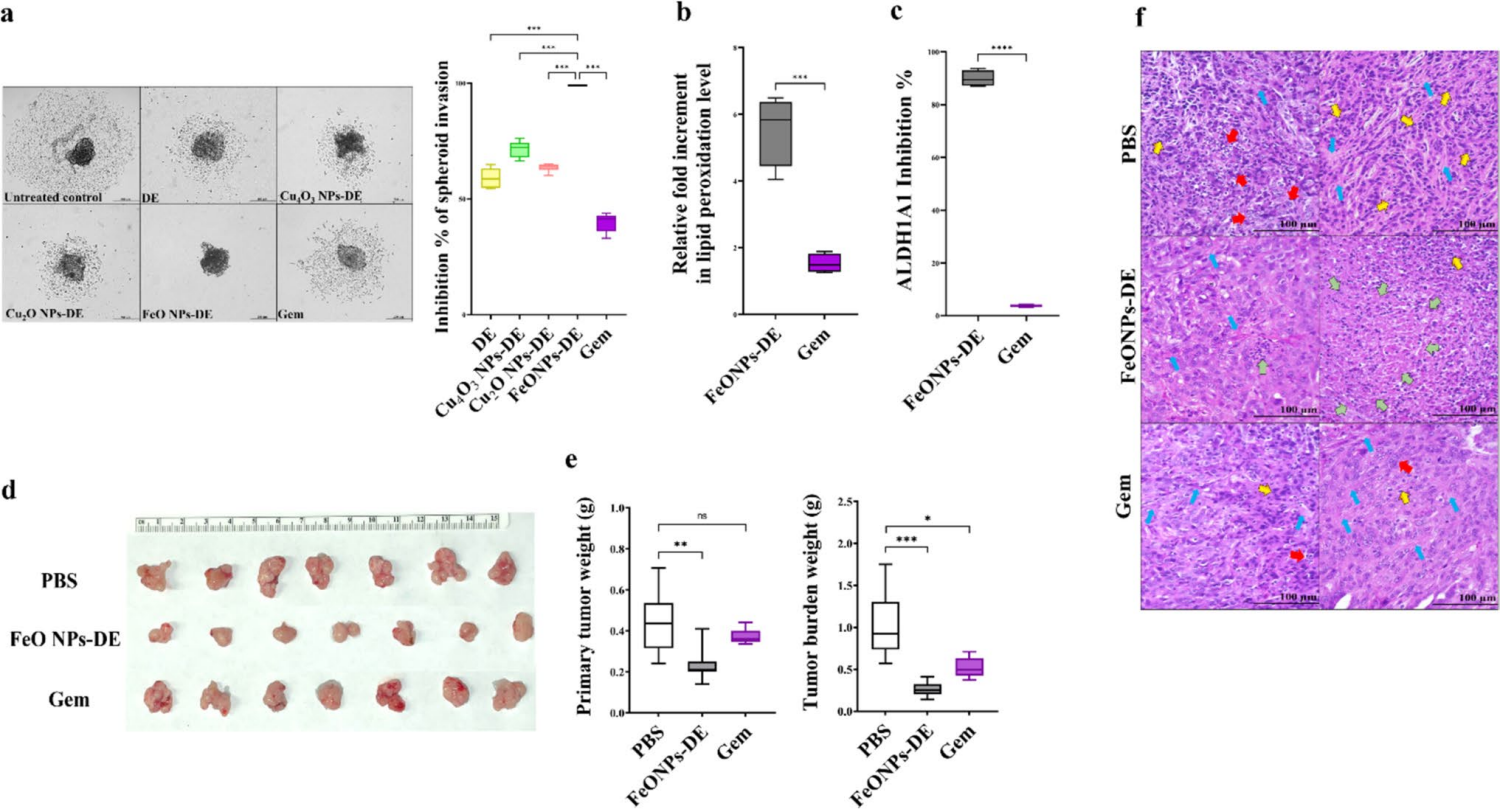
Invasion suppression, aldehyde dehydrogenase (ALDH)1A1 inhibition, and lipid peroxides elevation in the treated 1KPC:5PSC co-spheroids (in vitro 3D model) as well as tumor reduction and histological analysis of the treated tumor-bearing mice (in vivo orthotopic 3D co-spheroid model). **a** Invasion inhibition effect of diethyldithiocarbamate (DE), nanocomplexes (Cu_4_O_3_ NPs-DE, Cu_2_O NPs-DE, and FeO NPs-DE), and standard chemotherapy (gemcitabine, Gem) as demonstrated by microscopic images (40 × magnification) and invasion inhibition percentages. Data presented as mean ± SEM. The nanocomplex of FeO NPs-DE was compared to other tested compounds and values are considered statistically significant at *p* < 0.05*, < 0.001**, and < 0.0001***. **b** Fold increment in lipid peroxidation level and **c** ALDH1A1 activity inhibition in the treated co-spheroids relative to the untreated co-spheroids. Data presented as mean ± SEM. The nanocomplex of FeO NPs-DE was compared to Gem and values are considered statistically significant at *p* < 0.05*, < 0.001**, and < 0.0001***. **d** Image of harvested primary PDAC tumor tissues of phosphate buffer saline (PBS)-treated, FeO NPs-DE-treated, and Gem-treated tumor-bearing C57BL/6J mice. **e** Weights of primary tumor and total tumor burden (primary plus peritoneal metastatic tumor) for all studied mouse groups. Data presented as mean ± SEM. PBS-treated mouse group compared to FeO NPs-DE and Gem groups. Values are considered statistically significant at *p* < 0.05*, < 0.001**, and < 0.0001***. **f** Hematoxylin and eosin-stained tumor sections demonstrating hyperchromatic nuclear tumor cells (yellow arrows), polynuclear tumor cells (red arrows), and dead tumor cells (green arrows) as well as fibrotic stroma (blue arrows)

When 1KPC:5PSC spheroids were treated with 74 µg/mL of FeO NPs-DE or Gem (Fig. 5d), FeO NPs-DE-treated co-spheroids showed a greater reduction in the co-spheroid count (42.5%) and area (79.8%) than those treated with Gem (28.7% and 20.7%, respectively). Moreover, FeO NPs-DE suppressed the gene expression (CD24, CD44, CD133, ABCG2, ALDH1A1, OCT-4, TGF-β, COL1A2, SMA, FN, HIF-1α, and MMP9) more effectively than Gem (Fig. 5e). Interestingly, FeO NPs-DE completely inhibited the invasion potential of the co-spheroid, compared to 40%, 71.5%, 63.6%, and 39.8% for DE, Cu_4_O_3_ NPs-DE, Cu_2_O NPs-DE, and Gem, respectively (Fig. 6a).

Regarding alterations in the cellular redox status, Fig. 6b illustrates the significant elevation in lipid peroxidation (ferroptotic marker) levels (>5-fold) in FeO NPs-DE-treated co-spheroids relative to Gem-treated co-spheroids. FeO NPs-DE inhibited ALDH1A1 activity by 89.6%, whereas Gem repressed it by only 4.04%, as shown in Fig. 6c.

### In vivo therapeutic potency of FeO NPs-DE using the orthotopic 3D model for PDAC:

#### Tumor weight and histological analysis

To determine the ideal number of co-spheroids and initiation of treatment, different numbers of co-spheroids were tested. Therefore, orthotopic injections of 10, 50, or 100 co-spheroids were performed. By injecting 100 co-spheroids, tumors developed (0.26 g) 7 days after tumor implantation. Meanwhile, the injection of 50 co-spheroids resulted in a similar tumor weight (0.22 g) after 14 days, and the administration of 10 co-spheroids did not generate any tumors during the 14 days. Neither injection of 50 nor 10 co-spheroids generated peritoneal tumors after 7 and 14 days, respectively. There was no difference between the H&E-stained tumor sections of 100 co-spheroids (7th day) and 50 co-spheroids (14th day), showing hyperchromatic nuclei of poorly differentiated tumor cells with dense fibrotic stroma (Supplementary Fig. 2a,b). Therefore, the therapeutic efficiency of the most active nanocomplex (FeO NPs-DE) was evaluated after 7 days after injection of 100 co-spheroids by treating tumor-bearing mice with FeO NPs-DE for 3 weeks and comparison with Gem. FeO NPs-DE reduced the primary tumor weight by approximately 47%, whereas a reduction of 15.9% was observed in Gem-treated tumor-bearing mice (Fig. 6d,e). Notably, 71% of the FeO NPs-DE-treated tumor-bearing mice did not have peritoneal metastatic tumors, whereas these appeared in all Gem-treated tumor-bearing mice (Supplementary Fig. 2c). As demonstrated in Fig. 6e, the weights of total tumor burdens in PBS-treated mice, FeO NPs-DE-treated mice, and Gem-treated mice were 1.05±0.15, 0.26±0.03, and 0.52±0.04 g, respectively.

The aggressive tumor stage was confirmed by increasing areas of poorly differentiated polynucleated cells (red arrows) and hyperchromatic nuclear tumor cells (yellow arrows), as well as extensive fibrotic stroma (blue arrows) in H&E-stained tumor sections from PBS-treated tumor-bearing mice (Fig. 6f). Histological analysis of the FeO NPs-DE-treated group demonstrated larger sites of dead cells (green arrows), with smaller areas of poorly differentiated tumor cells (yellow arrow) and less extensive fibrotic stroma (blue arrows) than those in the Gem-treated group. The latter showed fewer areas of polynucleated tumor cells (red arrows) and hyperchromatic nuclear cells (yellow arrows) than in the PBS-treated group (Fig. 6f).

#### Main markers of CSCs and the activated PSCs

**Fig. 7:**
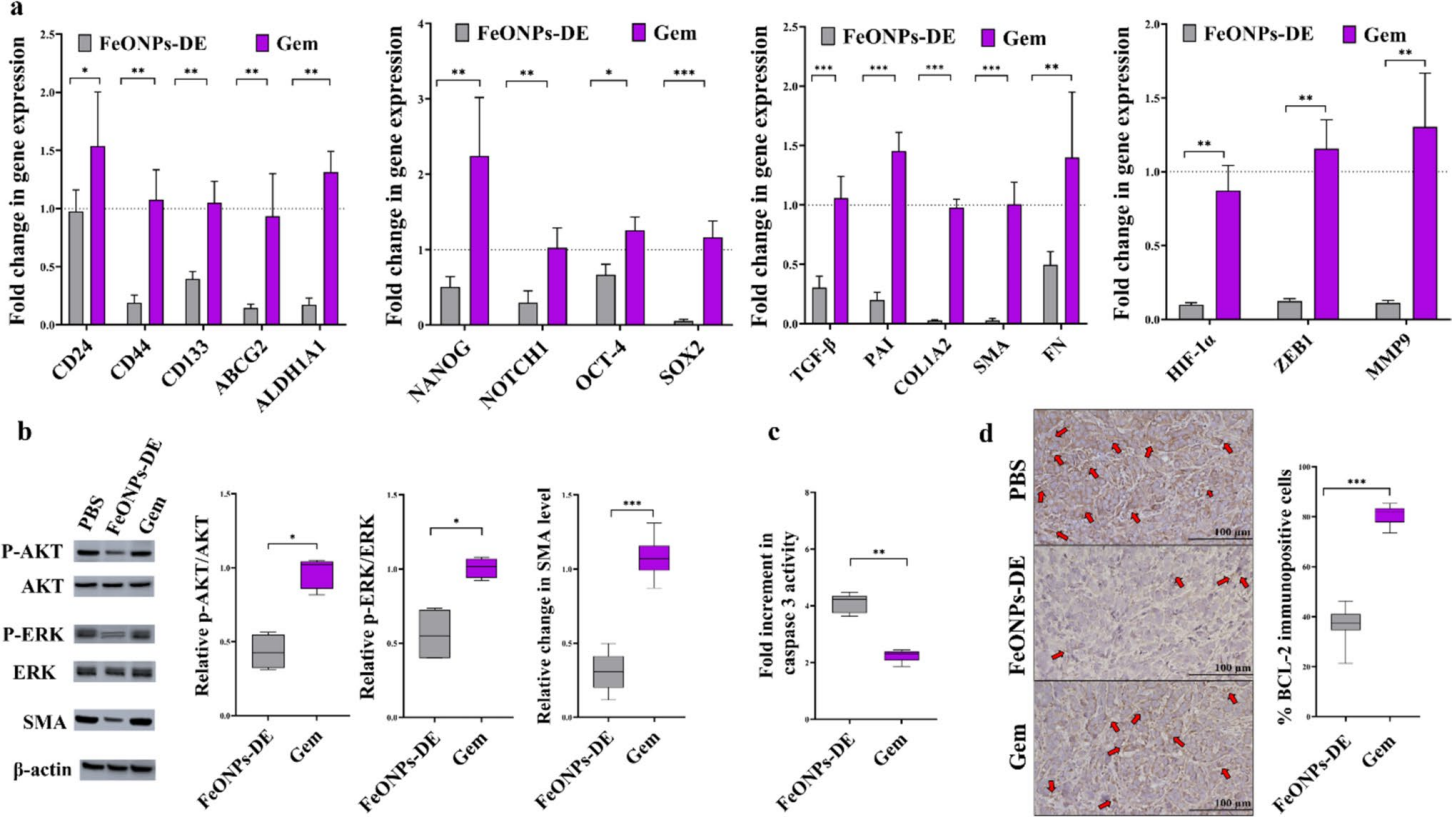
Suppressive effects on the main gene and protein expressions of cancer stem cells (CSCs) and pancreatic stellate cells (PSCs) as well as alteration in pro-apoptotic and anti-apoptotic markers in the treated tumor-bearing mice. **a** Relative fold decrease in the gene expression of CD24, CD44, ATP-binding cassette drug transporter (ABC)G2, aldehyde dehydrogenase (ALDH)1A1, NANOG, NOTCH1, OCT-4, SOX2, collagen (COL)1A2, CD10, plasminogen activator inhibitor-1 (PAI), smooth muscle actin (SMA), hypoxia-inducing factor (HIF)-1α, ZEB1, and matrix metalloproteinase (MMP)9 in the treated tumor-bearing groups (FeO nanoparticles (NPs)-diethyldithiocarbamate (DE) and gemcitabine (Gem)). **b** Western blot analysis of p-AKT/AKT, phosphorylated extracellular signal-regulated kinase (p-ERK)/ERK, and SMA, shown by chemiluminescent blot images and densitometric bar graphs. **c** Fold increment in key pro-apoptotic marker (caspase 3 activity) in the treated tumor-bearing mouse groups. **d** Immunohistochemical analysis of anti-apoptotic protein (BCL-2) immunostaining, as shown by the brown color of BCL-2-positive immunostained tumor cells (red arrows) Data presented as mean ± SEM. The nanocomplex of FeO NPs-DE was compared to Gem. Values are considered statistically significant at *p* < 0.05*, < 0.001**, and < 0.0001***

Figure 7a shows that FeO NPs-DE significantly outperformed Gem in downregulating the gene expression of CSCs (CD24, CD44, and CD133) by 1.5-fold, activated PSC markers (PAI, TGF-β, COL1A2, FN, and SMA) by > twofold, and activated their common stemness genes (ABCG2, ALDH1A1, NANO, NOTCH1, OCT-4, SOX2, HIF-1α, ZEB1, and MMP9) by ≥ twofold.

More importantly, western blot analysis (Fig. 7b) revealed that FeO NPs-DE significantly attenuated the phosphorylation of AKT and ERK, as evidenced by the reducing the relative ratios of p-AKT/AKT and p-ERK/ERK by 1.8-fold and 2.3-fold, respectively, compared with Gem. Furthermore, western blot analysis of SMA (overexpressed in activated PSCs) illustrated that FeO NPs-DE had a higher diminishing potency (3.26-fold) than Gem (Fig. 7b).

#### Apoptosis and tumorigenesis markers

Caspase 3 activity was enhanced in FeO NPs-DE-treated and Gem-treated mice by 4.1-fold and 2.4-fold, respectively, relative to that in the PBS-treated group (Fig. 7c). IHC analysis of the anti-apoptotic marker (BCL-2) declared that the percentages of BCL-2-immunostained tumor cells (red arrows) in FeO NPs-DE-treated and Gem-treated mice were 37.6 ± 0.75% and 80.6 ± 0.55%, respectively, relative to the PBS-treated group (Fig. 7d).

#### Selective tumoral uptake, ferroptosis induction, and ALDH1A1 inhibition

**Fig. 8:**
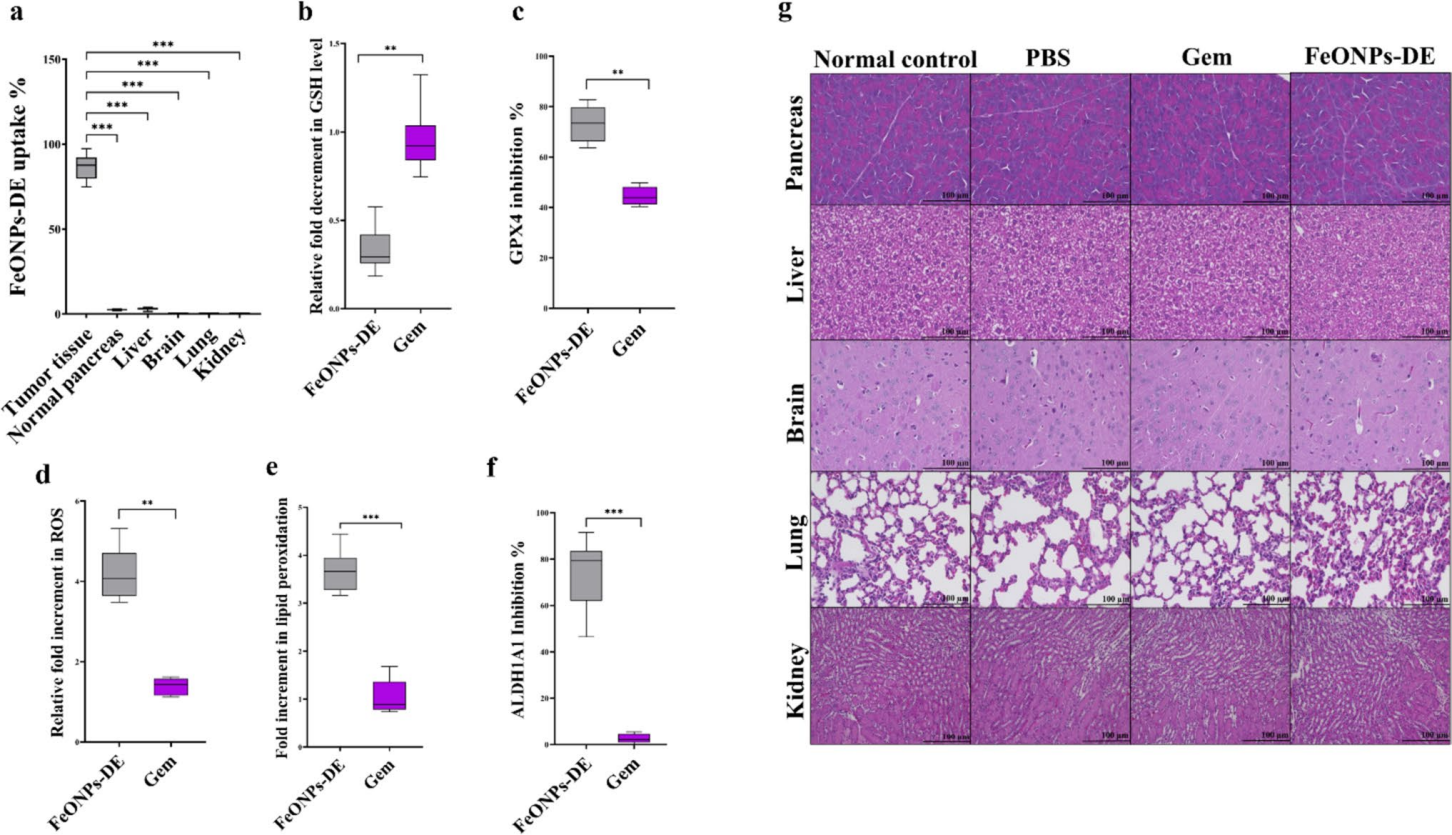
Alteration impact on ferroptosis markers and inhibitory effect on aldehyde dehydrogenase (ALDH)1A1 activity as well as histological analysis (hematoxylin and eosin staining) of normal tissue sections. **a** Uptake percentage of FeO nanoparticles (NPs)-diethyldithiocarbamate (DE) in tumor and normal tissues, **b,c** anti-ferroptosis suppression, including fold decrement in the reduced glutathione (GSH) level and glutathione peroxidase (GPX)4 activity inhibition, respectively, and **d,e** elevation of ferroptosis markers (reactive oxygen species (ROS) content and lipid peroxidation level, respectively). **f** ALDH1A1 inhibition percentage in FeO NPs-DE- and gemcitabine (Gem)-treated mouse groups relative to the PBS-treated group. Data presented as mean ± SEM. The nanocomplex of FeO NPs-DE nanocomplex was compared to Gem. Values are considered statistically significant at *p* < 0.05*, < 0.001**, and < 0.0001***. **g** Hematoxylin and eosin staining of the untreated and treated normal tissue sections

Ferroptosis is triggered by intracellular iron accumulation. Therefore, iron levels were assessed in different tissues. It was found that the tumoral uptake of FeO NPs-DE was 86.59 ± 2.15%, compared to only 2.49 ± 0.16% and 2.97 ± 0.25% absorbed by the normal pancreas and liver tissues, respectively (Fig. 8a). Meanwhile, no uptake of FeO NPs-DE was observed in other normal tissues. Moreover, FeO NPs-DE exhibited a stronger potential than Gem for lowering anti-ferroptotic antioxidant markers (GSH and GPX4) by 2.9-fold and 1.6-fold, respectively, and for enhancing ferroptosis markers (ROS and lipid peroxidation) by 3.1-fold and 3.6-fold, respectively (Fig. 8b–e). In FeO NPs-DE-treated tumor-bearing mice, this nanocomplex had a higher potency for inhibiting ALDH1A1 activity by 74.2 ± 4.04% compared to Gem-treated tumor-bearing mice (2.59 ± 0.56%), as shown in Fig. 8f. The safety of this nanocomplex and Gem was also ascertained by normal histology of these normal tissues (pancreas, liver, brain, lung, and kidney) compared with untreated control healthy and PBS-treated mice (Fig. 8g).

## Discussion

The death rate of PDAC, rather than other cancer types, has remained high despite advanced research efforts in recent years [35]. This high mortality is linked to high therapeutic resistance and metastatic potential that are primarily attributable to the stemness characteristics of CSCs and PSCs [2, 3]. Current drugs (e.g., Gem) preferentially target differentiated cancer cells, leaving CSCs, and their delivery is blocked by activated PSCs. Therefore, there is an urgent need to improve existing treatments and generate novel CSC- and PSC-targeted therapeutic strategies [3, 9, 24]. Accordingly, a 3D co-spheroid model was established in this study by optimizing the ratio of KPC to PSC spheroids (1:5). This KPC:PSC ratio exhibited the highest invasive potential and gene expression levels of CSC and activated PSC markers, compared to other ratios. Previous studies have reported direct cross-talk between human cancer cells (Panc1) and PSCs, in 3D co-spheroids (1:1), by higher gene expression of PSC activation markers (TGF-β, Col1, SMA, and FN) than in 2D model and mono-spheroids [1, 9]. In this co-spheroid model, the excessive expression of extracellular matrix proteins mediated hypoxia and drug resistance, subsequently leading to stemness [1, 9, 36, 37]. During spheroid proliferation, the depletion of oxygen (hypoxia), nutrients, and pH increase toward the inner layers of spheroids, coupled with the deposition of extracellular matrix proteins, generating a physical barrier that limits drug efficacy. Therefore, the 3D co-spheroid model more accurately resembles the stromal environment, architecture, stroma–cancer interactions, and stemness behavior of a clinical tumor than the 2D model [9, 13].

As PDAC cells are resistant to the apoptotic effect of traditional chemotherapeutic drugs, the discovery of an efficient non-apoptotic therapeutic strategy (e.g., cuproptosis and ferroptosis) is of critical importance [38]. Herein, FeO NPs-DE showed the highest cytotoxicity against Panc-1, MIA PaCa-2, and KPC (IC_50_ ≤ 8.21 µg/mL) and anti-migration activity, as well as the strongest inhibition for mono- and co-spheroid growth (IC_50_ < 78 and ~ 74 µg/mL, respectively) with low cytotoxicity against PSC spheroids. In these treated human and mouse PDAC mono-spheroids, stemness gene expression levels and ALDH1A1 activity were inhibited and lipid peroxidation content elevated significantly, indicating that ferroptosis-mediated stemness inhibition effect was induced in both human and mouse PDAC spheroids (Fig. 3a,b**)**. Furthermore, FeO NPs-DE inhibited the invasion of KPC-PSC spheroids more effectively than other compounds (copper oxide-DE nanocomplexes, DE, and Gem). In FeO NPs-DE-treated co-spheroids (Fig. 5c), the underpinning cell death mode is primarily ferroptosis and secondarily apoptosis as evidenced by a higher viability of FeO NPs-DE + Fer-1-treated co-spheroids than FeO NPs-DE + Z-VAD-FMN-treated co-spheroids. The strongest efficacy of FeO NPs-DE, as ascertained by the significant downregulation of CSC and PSC genes, is mainly attributed to the induction of ferroptosis-dependent lipid peroxidation, which is potentiated by DE activity for inhibiting ALDH1A1-mediated lipid peroxide detoxification (Fig. 3b, 5e, 6b, 6c). Moreover, FeO NPs-DE exhibited a higher tumor-eradicating effect than Gem using in vivo co-spheroid model (Fig. 6–8). The low sensitivity of PDAC to apoptotic and pro-oxidant activities of Gem is primarily linked to the ABCG2-dependent drug efflux capacity of CSCs and PSCs, as well as the stroma, which reduces drug delivery [2, 3, 39]. Furthermore, stemness promotes Gem-resistance-mediated anti-apoptosis via activation of the AKT pathway and a highly efficient antioxidant system (glutathione and ALDH1A1) [39]. CSC markers (CD24, CD44, and CD133) act as positive regulators of stemness factor-mediated chemoresistance (NANOG, NOTCH1, OCT-4, SOX2, HIF-1α, ABCG2, and ALDH). CD44 and CD133 also upregulate key antioxidant regulators (GSH and ALDH1, respectively) and PSC activators (p-AKT, p-ERK, and ZEB1) [3, 8, 9]. Therefore, the spheroid model is more resistant than the 2D model to the pro-oxidant and DNA-damaging effects of Gem [39, 40].

On the other hand, stemness signatures protect tumor from ferroptosis [41]. Ferroptosis (iron-dependent cell death) is manifested by loss of redox homeostasis and terminates with increasing lipid peroxidation-mediated membrane, DNA, and mitochondrial damage [42]. To selectively trigger ferroptosis in tumor cells without damaging other normal tissues, iron was administered as NPs, and it was confirmed by the highest accumulation percentage of FeO NPs-DE in tumor tissues (Fig. 8a) and normal histology of other healthy tissues (Fig. 8g). The selective accumulation of this nanocomplex (Fig. 8a) is mainly linked to the enhanced permeability and retention (EPR) phenomenon, which is based on abnormal tumoral vasculature and its nano-size (157.8 nm). The nanoparticles with a size of 100–200 nm are optimal for achieving the EPR effect and escaping from normal tissues (e.g., liver) [43]. In line with the author’s recent studies, FeO NPs-DE injection accumulated selectively in liver and mammary tumor tissues with no significant uptake by other normal tissues [15, 16]. The accumulated iron generates overproduction of ROS via the Fenton reaction, initiating lipid peroxidation, and the produced lipid radicals can trigger new chain reactions [44]. To execute ferroptosis efficiently without facing CSC resistance, DE was used. DE has thiol affinity to form disulfide adducts, which inhibits not only the glutathione system (GSH and GPX4) but also the robust stemness marker ALDH1 [12, 45, 46]. The latter is involved in the acquisition of stemness phenotypes, including self-renewal, ROS detoxifying-mediated apoptosis resistance, ABCG2 upregulating-mediated chemoresistance, and metastasis. ALDH1 generates retinoic acid, which activates the AKT and HIF-1α/vascular endothelial growth factor (VEGF) pathways, enhancing stemness and subsequently promoting aggressive tumor proliferation [13, 47, 48]. Suppressing AKT activation attenuates stemness (via downregulation of NANOG and SOX2), PSC activation, and BCL-2 overexpression, consequently triggering apoptosis [5, 49–51]. Moreover, DE exhibits pro-apoptotic activity via caspase activation [14, 52]. This indicates that the nanocomplex induced tumor cell death by DE-mediated (ALDH1A1 inhibition and caspase 3 activation) apoptosis as well as through the accumulation of Fe ONPs and DE-stimulated (lipid peroxidation overgeneration with suppressing antioxidant system) ferroptosis.

Accordingly, the selective accumulation of FeO NPs and DE generates excessive production of ROS and lipid peroxidation under glutathione system depletion and ALDH1A1 inhibition, resulting in lethal accumulation of lipid peroxidation with the collapse of CSCs and PSCs. The latter was ascertained by downregulating the gene expression of stemness, PSC activators, and PSC mediators (stromal proteins), as well as the protein levels of p-AKT, p-ERK, and the main stromal protein, SMA (Fig. 7a,b). Both stimulated cell death types (ferroptosis and apoptosis) were demonstrated as necrotic cells (right image) and cellular shrinkage (left image), respectively, in H&E-stained tumor sections of the FeO NPs-DE-treated mouse group (Fig. 6f). This indicates that DE mediated ferroptosis activation and stemness inhibition, as well as enhancing caspase 3-dependent apoptosis and lowering the protein level of the key oncogene, BCL-2 (Fig. 7c,d), and ultimately this nanocomplex significantly suppressed primary and secondary tumor growth.

## Conclusion

A 3D co-spheroid KPC:PSC (1:5) model, which is more similar to clinical PDAC tumors than the 2D model, was established and used to investigate the effect of FeO NPs-DE, other DE nanocomplexes, and Gem on the cellular components of CSCs and PSCs (key PDAC cellular components). In addition to in vitro 2D and 3D mono-spheroid models, the established 3D co-spheroid in vitro and in vivo models showed higher anti-PDAC efficacy of FeO NPs-DE than Gem. This potent effect is attributed to the selective accumulation of FeO NPs-DE in tumor tissues, inducing iron-dependent ferroptosis that was potentiated by the inactivation effect of DE on the glutathione system and ALDH1A1 activity-mediated stemness, as well as its apoptotic activity. This caused a significant collapse of CSCs and PSCs, subsequently halting PDAC growth. Accordingly, FeO NPs-DE may represent a new therapeutic strategy for the effective eradication of PDAC.

## Supplementary Information

Below is the link to the electronic supplementary material.

Supplementary Fig. 1 Morphology of PSC spheroids, KPC spheroids, and co-spheroids compared to 2D model. (a) PSC, KPC, and different ratios of KPC:PSC co-spheroids, in the terms of spheroid morphology (40x Magnification) and the estimated spheroid area. (b) Morphology of 2D and 3D models of PSC, KPC, and 1KPC:5PSC (100x magnification). Data are illustrated as mean±SEM. Spheroid area values were compared and considered statistically significant at p <0.05*, <0.001**, and <0.0001***.

Supplementary Fig. 2 In vivo induction trials of pancreatic cancer by orthotopic injection of 1KPC:5PSC co-spheroids and morphology of metastatic peritoneal tumors of different animal groups. (a) Histological analysis of tumor sections of mice that were injected with 10 co-spheroids, 50 co-spheroids, and 100 co-spheroids after 14, 14, and 7 days, respectively. (b) Tumor weights of two latter mouse groups. Data are illustrated as mean±SEM. Tumor weight values were compared and considered statistically significant at p <0.05*, <0.001**, and <0.0001***. (c) Image of harvested secondary (peritoneal) tumor tissues from phosphate buffer saline (PBS)-treated, FeO NPs-DE-treated, and Gem-treated tumor-bearing C57BL/6J mice that were induced by a single orthotopic injection of 100 co-spheroids/mouse, and after 7 days intraperitoneal injections of treatments were performed for 3 weeks (3 times/week).

Supplementary file 3 (DOCX 15 KB)

Supplementary file 4 (DOCX 16 KB)
