# Supplementary figures and images for: Ferroptosis- and stemness inhibition-mediated therapeutic potency of ferrous oxide nanoparticles-diethyldithiocarbamate using a co-spheroid 3D model of pancreatic cancer
Source: J Gastroenterol. 2025 Jan 31;60(5):641–57. doi: 10.1007/s00535-025-02213-3 (PMC12014774; doi:10.1007/s00535-025-02213-3)

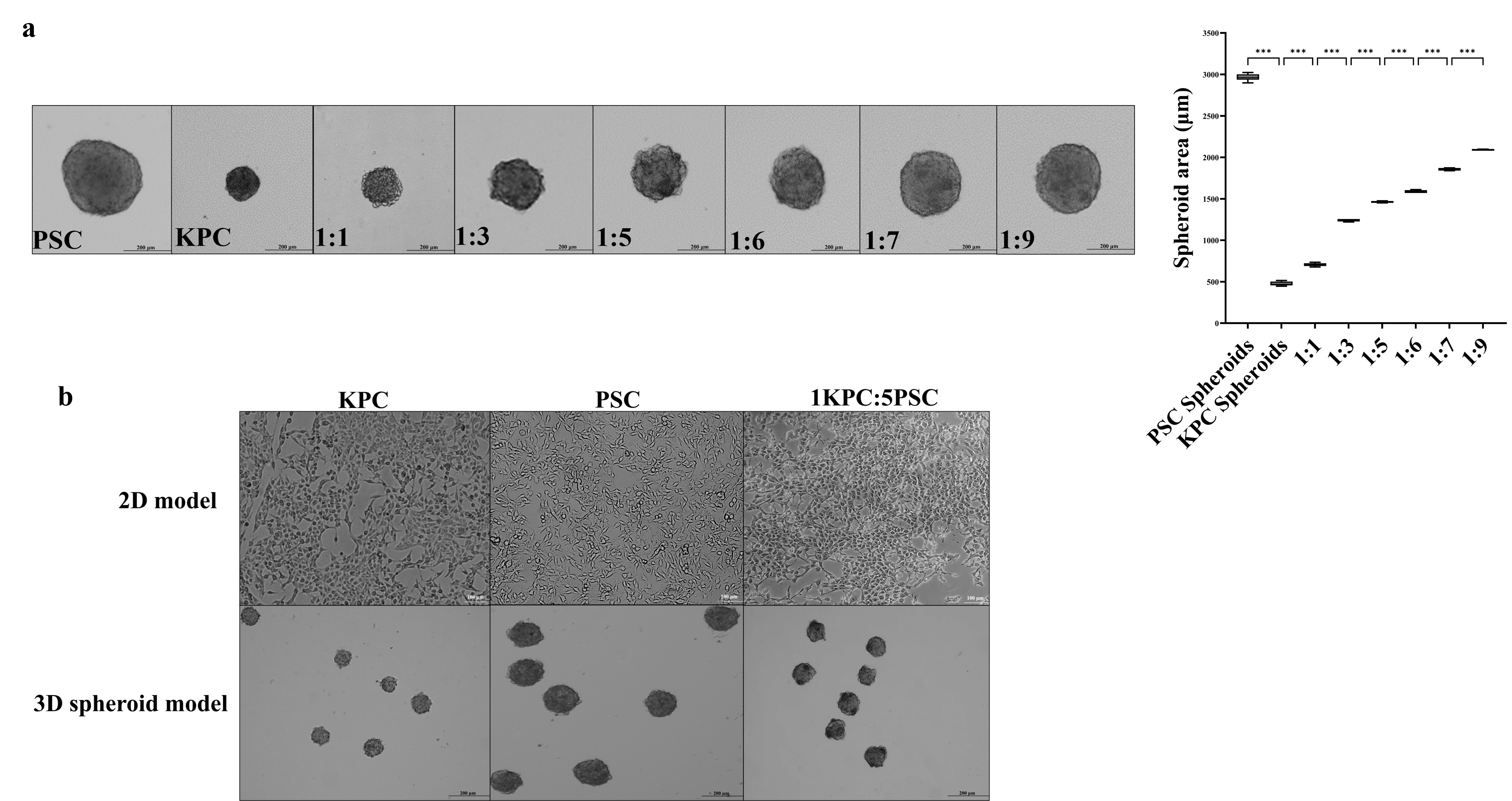

Supplement: Supplementary file 1 — Supplementary Fig. 1 Morphology of PSC spheroids, KPC spheroids, and co-spheroids compared to 2D model. (a) PSC, KPC, and different ratios of KPC:PSC co-spheroids, in the terms of spheroid morphology (40x Magnification) and the estimated spheroid area. (b) Morphology of 2D and 3D models of PSC, KPC, and 1KPC:5PSC (100x magnification). Data are illustrated as mean±SEM. Spheroid area values were compared and considered statistically significant at p <0.05*, <0.001**, and <0.0001***. [file 535_2025_2213_MOESM1_ESM.tif]

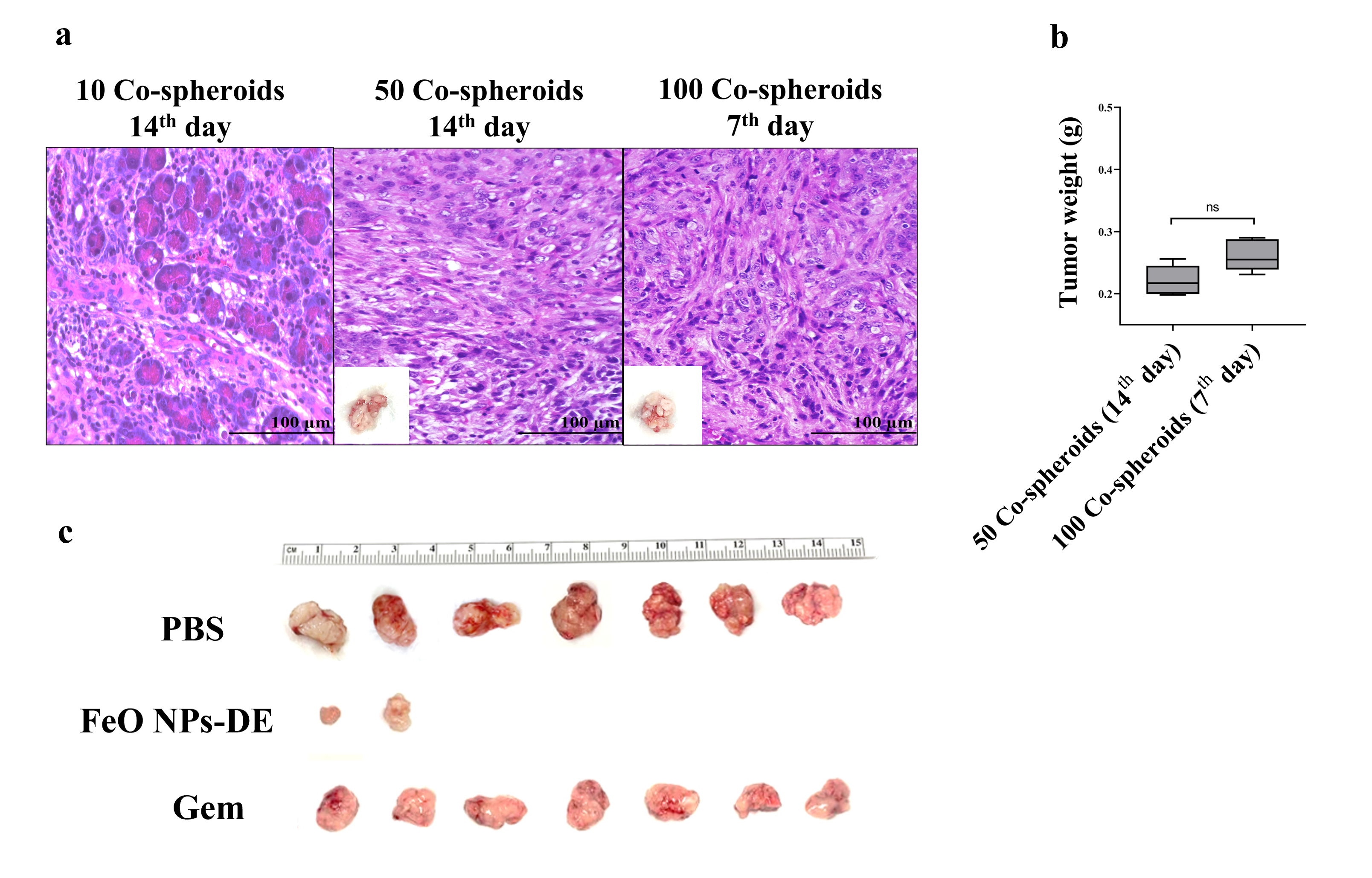

Supplement: Supplementary file 2 — Supplementary Fig. 2 In vivo induction trials of pancreatic cancer by orthotopic injection of 1KPC:5PSC co-spheroids and morphology of metastatic peritoneal tumors of different animal groups. (a) Histological analysis of tumor sections of mice that were injected with 10 co-spheroids, 50 co-spheroids, and 100 co-spheroids after 14, 14, and 7 days, respectively. (b) Tumor weights of two latter mouse groups. Data are illustrated as mean±SEM. Tumor weight values were compared and considered statistically significant at p <0.05*, <0.001**, and <0.0001***. (c) Image of harvested secondary (peritoneal) tumor tissues from phosphate buffer saline (PBS)-treated, FeO NPs-DE-treated, and Gem-treated tumor-bearing C57BL/6J mice that were induced by a single orthotopic injection of 100 co-spheroids/mouse, and after 7 days intraperitoneal injections of treatments were performed for 3 weeks (3 times/week). [file 535_2025_2213_MOESM2_ESM.tif]
