# Supplementary material for: Ferroptosis- and stemness inhibition-mediated therapeutic potency of ferrous oxide nanoparticles-diethyldithiocarbamate using a co-spheroid 3D model of pancreatic cancer
Source: J Gastroenterol. 2025 Jan 31;60(5):641–57. doi: 10.1007/s00535-025-02213-3 (PMC12014774; doi:10.1007/s00535-025-02213-3)
Supplement: Supplementary file 3 — (DOCX 15 KB) [file 535_2025_2213_MOESM3_ESM.docx]

**Ferroptosis- and stemness inhibition-mediated therapeutic potency of ferrous oxide nanoparticles-diethyldithiocarbamate using a co-spheroid 3D model of pancreatic cancer**

**Marwa M Abu-Serie^1*^, Ana K. Gutiérrez-García^2^, Macie Enman^2^, Utpreksha Vaish^2^, Huma Fatima^3^, Vikas Dudeja^2^**

^1^Medical Biotechnology Department, Genetic Engineering and Biotechnology Research Institute, (GEBRI), City of Scientific Research and Technological Applications (SRTA-City), New Borg El‑Arab City, Alexandria 21934, Egypt. **Phone:** +2034593422 **Fax:** +2034593407

*Correspondence: [marwaelhedaia@gmail.com](mailto:marwaelhedaia@gmail.com)

^2^Division of Surgical Oncology, Department of Surgery, University of Alabama at Birmingham (UAB), Birmingham, Alabama 35294, US

^3^Division of Anatomic Pathology, Department of Pathology, UAB, Birmingham, Alabama 35249, US.

**Supplementary Table 1** Human primer sequences (forward “F” and reverse “R”) of the used genes

| CD44 | F: GGGATATCGCCAAACACCCA  R: TGGATGGCTGGTATGAGCTG |
| --- | --- |
| ABCG2 | F: CTGTTTTGTGTTTATGATGGTCTGT  R: TGCTGCAAAGCCGTAAATCC |
| Aldehyde dehydrogenase (ALDH) 1A1 | F: TGGACCAGTGCAGCAAATCA  R: ACGCCATAGCAATTCACCCA |
| NANOG | F: AATGGTGTGACGCAGGGATG  R: TGCACCAGGTCTGAGTGTTC |
| NOTCH1 | F: CAGACTATGCCTGCAGCTGTG  R: CTGGCACGATTTCCCTGACC |
| OCT-4 | F: AGCACTTCTGTCATGCTGGA  R: AGCACCTTCTATAAGCCAGCG |
| SOX2 | F: CATGAAGGAGCACCCGGATT  R: TAACTGTCCATGCGCTGGTT |
| β-actin | F: GTCATTCCAAATATGAGATGCGT  R: GCTATCACCTCCCCTGTGTG |
