# Supplementary material for: Ferroptosis- and stemness inhibition-mediated therapeutic potency of ferrous oxide nanoparticles-diethyldithiocarbamate using a co-spheroid 3D model of pancreatic cancer
Source: J Gastroenterol. 2025 Jan 31;60(5):641–57. doi: 10.1007/s00535-025-02213-3 (PMC12014774; doi:10.1007/s00535-025-02213-3)
Supplement: Supplementary file 4 — (DOCX 16 KB) [file 535_2025_2213_MOESM4_ESM.docx]

**Ferroptosis- and stemness inhibition-mediated therapeutic potency of ferrous oxide nanoparticles-diethyldithiocarbamate using a co-spheroid 3D model of pancreatic cancer**

**Marwa M Abu-Serie^1*^, Ana K. Gutiérrez-García^2^, Macie Enman^2^, Utpreksha Vaish^2^, Huma Fatima^3^, Vikas Dudeja^2^**

^1^Medical Biotechnology Department, Genetic Engineering and Biotechnology Research Institute, (GEBRI), City of Scientific Research and Technological Applications (SRTA-City), New Borg El‑Arab City, Alexandria 21934, Egypt. **Phone:** +2034593422 **Fax:** +2034593407

*Correspondence: [marwaelhedaia@gmail.com](mailto:marwaelhedaia@gmail.com)

^2^Division of Surgical Oncology, Department of Surgery, University of Alabama at Birmingham (UAB), Birmingham, Alabama 35294, US

^3^Division of Anatomic Pathology, Department of Pathology, UAB, Birmingham, Alabama 35249, US.

**Supplementary Table 2** Mouse primer sequences (forward “F” and reverse “R”) of the used genes

| CD24 | F: TGCTTGATGCAGCACTAGAGG  R: GCCCCAGCCGTGAGTATG |
| --- | --- |
| CD44 | F: GATGCCTGTTGCAAGTACTCC  R: AGCACTGGTGCTGATTCTGT |
| CD133 | F: AATTAAGTGGAAGGAGCCCAG  R: TCTCCAAGGTGGTCATTCACT |
| ABCG2 | F: TGAATAGCTGACCATCAGTGCC  R: GCTGCTTAATCTGGCCTCACA |
| Aldehyde dehydrogenase (ALDH) 1A1 | F: TAAAGCTGGGAAATGCCCCC  R: TGCAAACCTCTCCCTTTGCT |
| NANOG | F: GGCTCACTTCCTTCTGACTTCT  R: CTCATGTCAGTGTGATGGCG |
| NOTCH1 | F: CACCAGGGTGGTCAGGAAAA  R: GGGCAGCGACAGATGTATGA |
| OCT-4 | F: GGGCTAGAGAAGGATGTGGTTC  R: GAAAGGTGTCCCTGTAGCCTC |
| SOX2 | F: GGAGGAGAGCGCCTGTTTTT  R: CTGGCGGAGAATAGTTGGGG |
| Transforming growth factor (TGF)-β | F: CAACCCAGGTCCTTCCTAAA  R: GGAGAGCCCTGGATACCAAC |
| Plasminogen activator inhibitor-1 (PAI) | F: ACGCCTTCATTTGGGACGAA  R: GTCTGGGATGCTGGTTGGAA |
| Collagen (COL) 1A2 | F: TAGGCCATTGTGTATGCAGC  R: ACATGTTCAGCTTTGTGGACC |
| Smooth muscle actin (SMA) | F: GTTCAGTGGTGCCTCTGTCA  R: ACTGGGACGACATGGAAAAG |
| Fibronectin (FN) | F: TGGTGGCCACTAAATACGAA  R: GGAGGGCTAACATTCTCCAG |
| Hypoxia inducing factor (HIF)-1α | F: AGGATGAGTTCTGAACGTCGAAA  R: GGGGAAGTGGCAACTGATGA |
| ZEB1 | F: TCCCCAGCTCACAATAAACG  R: GCTCTAGGATGTAATGCCCAC |
| Matrix metalloproteinase (MMP) 9 | F: TCCTTGCAATGTGGATGTTT  R: CTTCCAGTACCAACCGTCCT |
| CXCR4 | F: CATGGAACCGATCAGTGTGAG  R: AAGCAGGGTTCCTTGTTGGA |
| 18s rRNA | F: GCAATTATTCCCCATGAACG  R: GGCCTCACTAAACCATCCAA |
